# Biologics, cardiovascular effects and cancer

**DOI:** 10.1186/1741-7015-12-48

**Published:** 2014-03-18

**Authors:** Nemanja Damjanov, Michael T Nurmohamed, Zoltán Szekanecz

**Affiliations:** 1 Belgrade University School of Medicine, Belgrade, Serbia; 2 VU University Medical Center & Jan van Breemen Research Institute, Amsterdam, The Netherlands; 3 Department of Rheumatology, Institute of Medicine, University of Debrecen, Faculty of Medicine, Debrecen, Hungary

**Keywords:** Rheumatoid arthritis, TNF-inhibitors, Cardiovascular risk, Cancer risk, Malignancies

## Abstract

Rheumatoid arthritis (RA) is associated with increased cardiovascular risk. Treatment with tumor necrosis factor (TNF)-inhibitors leads to about a 50% reduction in the first cardiovascular event. TNF-inhibitors could transiently improve flow-mediated vasodilation and improve carotid intima-media thickness (ccIMT) during the treatment of RA. Treatment with TNF-inhibitors is associated with an increased total cholesterol (TC) and HDL-cholesterol (HDLc) level, without sustained change of the atherogenic index. The overall cancer risk in RA patients is comparable to that of the general population, but patients with RA slightly more often have lymphomas and lung tumors, and less often have colorectal and breast tumors in comparison to the general population. In randomized controlled trials (RCT) TNF-inhibitors did not increase the risk of solid malignancies, except for non-melanoma skin cancer (risk doubled compared to control treatment). Meta-analysis of registries and long-term extension studies showed no increased risk for total malignancies as well as for non-melanoma skin cancer when comparing TNF-inhibitors and the classical disease modifying anti-rheumatic drugs (DMARDs) treatment.

## Background

Rheumatoid arthritis (RA) is associated with an approximately doubled cardiovascular risk that approaches that of diabetes. There is accumulating evidence that biologics, particularly TNF-inhibitors, reduce the cardiovascular risk in RA [1,2]. This might be mediated through favorable effects on the vasculature and/or the lipid profile.

Another clinically important question is if, and to what extent, biologics increase the cancer risk in RA. Since it is well known that lymphomas and lung tumors are more often present in RA patients, compared to the general population, it is important to know whether treatment with TNF-inhibitors increases the relative risk for malignancies in patients with RA.

### TNF-inhibitors and cardiovascular risk

One of the first studies investigating the effect of TNF-inhibitors on cardiovascular risk comes from Jacobsson *et al*. in 2005 [1]. Treatment with TNF-inhibitors led to a more than 50% reduction of first cardiovascular events. In the following years the findings of Jacobsson *et al*. were confirmed by other groups.

The British Society for Rheumatology Biologics Register comprises RA patients with active disease treated with TNF-inhibitors or DMARDs who are followed prospectively [2]. Remarkably, in the 2007 publication of this registry with almost 11,000 patients, there was no significant difference between the two groups when looking at incident myocardial infarction. However, when comparing the myocardial infarction rate between responders and non-responders to TNF-inhibitors, there was a more than 60% reduction in the rate of myocardial infarctions in the responding patients.

### Biologics and vascular function

Ultrasound-based techniques have been widely used to detect arterial endothelial dysfunction, overt carotid atherosclerosis and arterial stiffness by assessing flow-mediated vasodilation (FMD), common carotid intima-media thickness (ccIMT) and pulse-wave velocity (PWV)/augmentation index (AIx), respectively [3]. TNF-inhibitors, such as infliximab (IFX), etanercept (ETN) or adalimumab (ADA) improved FMD in numerous studies [4]. Most of these studies were short-term (12 to 36 weeks). At least in two cohorts, the favorable effects of biologics on FMD were transient, when endothelial dysfunction returned post-treatment [5,6]. Controversies have been observed with respect to ccIMT and stiffness assessments. Carotid atherosclerosis was beneficially influenced by 12 months of IFX treatment in established RA [7]. ADA also improved ccIMT in an early RA cohort [8]. On the other hand, no effects of biologics on ccIMT were observed in either cohort [4]. Anti-TNF therapy improved PWV but did not affect AIx in RA patients [4]. Thus, it is still uncertain whether biologics improve vascular function in RA or not.

### Biologics and lipid profile

Although nowadays there is convincing evidence that treatment with TNF-inhibitors is associated with a reduced cardiovascular risk, some argue that TNF-blocking therapy has adverse effects on the lipid profile that might translate into an increased cardiovascular risk instead of a decreased cardiovascular risk. As the literature appears contradictory in this respect several meta-analyses have been done. The first systematic review and meta-analysis comprised 15 studies encompassing 766 RA patients fulfilling the inclusion criteria [9]. This meta-analysis revealed an increased total cholesterol (TC) level (maximum increase of 10%), that leveled off after one year of therapy. HDL-cholesterol (HDLc) increased significantly in the first two to six weeks of therapy (maximum increase 7%) and decreased slightly after fifteen weeks of therapy. Thus, treatment with TNF-inhibitors has a considerable, albeit transient, effect on TC and HDLc levels in RA patients. There was no sustained improvement of the atherogenic index. Hence, the favorable effect of TNF-alpha blocking agents on the cardiovascular risk in RA is not mediated by beneficial effects on lipid metabolism. It is important to realize that the effects of biologics on lipids should be assessed in the phase in which patients have low disease activity in order to avoid the ‘lipid paradox’ [10].

### Rheumatoid arthritis and malignancies

The overall cancer risk in RA is comparable with the general population [11]. However, patients with RA more often have lymphomas and lung tumors with standardized incidence ratios of 2.1 and 1.6, respectively. It is important to realize that the risk for lymphoma is dependent on the disease activity, the higher the disease activity, the higher the chance for lymphomas. In contrast, patients with RA do have colorectal and breast tumors less often in comparison to the general population.

### TNF-inhibitors and cancer risk

Important information comes from the meta-analysis that was published at the end of 2010 following a request by the European Medicines Agency to the market authorization holders of the TNF-inhibitors to conduct a joint meta-analysis of their RCT-data [12]. A total of 74 trials with 22,000 patients either treated with adalimumab, etanercept or infliximab were included. Thirty-one of these studies were in RA and 43 in other indications. In total there were 178 malignancies, 130 in the TNF-inhibitors group and 48 in the controls. When excluding non-melanoma skin cancer, the relative risk was about 1, indicating that TNF-inhibitors do not increase the risk for solid malignancies. However, when only looking at non-melanoma skin cancer, the risk is approximately doubled when comparing treatment with TNF-inhibitors and control treatment. Obviously, this study indicates that TNF-blockade does, in the short term, not increase the risk for solid malignancies but obviously long-term risk assessment requires observational studies and registries.

A first meta-analysis of registries and long-term extension studies was published in 2012; after an extensive literature search these investigators identified twelve registries and five long-term extension studies [13]. When comparing TNF-inhibitors versus the classical DMARDs this meta-analysis indicated no increased risk for total malignancies or for non-melanoma skin cancer.

### Clinical consequences

As thus far the literature indicates no increased risk for solid malignancies, screening and testing for solid malignancies is not necessary when administering TNF-inhibitors. However, regular attention to the skin remains necessary when giving TNF-inhibitors, as the risk for non-melanoma skin cancer, particularly in combination with methotrexate, is approximately doubled. In this respect, it is also important to realize that a recent study indicated a 50% increased relative risk of invasive melanoma [14]. These authors indicate that given the small increase in absolute risk, the overall risk-benefit balance of TNF inhibitors remains favorable. However, in patients having an elevated risk for melanoma development, these drugs might be contraindicated.

## Conclusions

TNF-inhibitors reduce cardiovascular risk by about 50%. With respect to malignancies, these drugs probably are associated with an increased risk for non-melanoma and melanoma skin cancer making regular skin inspection necessary. The risk for solid malignancies appears not to be increased. Presently, the effects of TNF-inhibitors appear favorable both from a cardiovascular point of view as well as from a safety point of view, particularly when considering the substantial improvement in the quality of life associated with the use of these agents. However, as some of the evidence is still debatable, long-term prospective studies are still needed (and actually ongoing) to determine the risk of cardiovascular diseases and malignancies in biologic-treated arthritis patients.

## Abbreviations

ADA: adalimumab; Aix: Augmentation Index; ccIMT: carotid intima-media thickness; DMARDs: disease modifying anti-rheumatic drugs; ETN: etanercept; FMD: flow-mediated vasodilation; HDLc: HDL-cholesterol; IFX: infliximab; PWV: pulse-wave velocity; RA: rheumatoid arthritis; RCT: randomized controlled trials; TC: total cholesterol; TNF: tumor necrosis factor.

## Competing interests

The authors declare that they have no competing interests.

## Authors’ contributions

ND has made substantial contributions to the conception and design as well as interpretation of data presented in the manuscript, has been involved in drafting the manuscript and revising it critically for important intellectual content, has given final approval of the version to be published, and agrees to be accountable for all aspects of the work in ensuring that questions related to the accuracy or integrity of any part of the manuscript are appropriately addressed and resolved. MTN has made substantial contributions to the conception and design as well as interpretation of data presented in the manuscript, has been involved in revising it critically for important intellectual content, has given final approval of the version to be published, and agrees to be accountable for all aspects of the work in ensuring that questions related to the accuracy or integrity of any part of the manuscript are appropriately addressed and resolved. ZS has made substantial contributions to the conception and design as well as interpretation of data presented in the manuscript, has been involved in revising it critically for important intellectual content, has given final approval of the version to be published, and agrees to be accountable for all aspects of the work in ensuring that questions related to the accuracy or integrity of any part of the manuscript are appropriately addressed and resolved. All authors read and approved the final manuscript.
